# Differences in perception of breast cancer treatment between patients, physicians, and nurses and unmet information needs in Japan

**DOI:** 10.1007/s00520-019-05029-z

**Published:** 2019-09-03

**Authors:** Haruna Sakai, Megumi Umeda, Hiromi Okuyama, Seigo Nakamura

**Affiliations:** 1 Division of Breast Surgery, Department of Surgery, School of Medicine, Showa University, 1-5-8 Hatanodai Shinagawa-ku, Tokyo, 142-8555 Japan; 2 Division of Adult Nursing, Department of Nursing, Graduate School of Health Sciences, Showa University, 1-5-8 Hatanodai Shinagawa-ku, Tokyo, 142-8555 Japan; 3 Department of Clinical Pharmacy, Division of Drug Information Analytics, School of Pharmacy, Showa University, 1-5-8 Hatanodai Shinagawa-ku, Tokyo, 142-8555 Japan

**Keywords:** Breast cancer, Drug treatment, Adverse events, Decision-making, Patient needs

## Abstract

**Purpose:**

Discrepancies exist between healthcare provider and patient perceptions surrounding breast cancer treatment. Significant treatment changes in the last 10 years have made re-evaluation of these perceptions necessary.

**Methods:**

Physicians and nurses involved in breast cancer treatment, and patients who had received breast cancer chemotherapy (past 5 years), were questioned using an Internet survey. Participants ranked physical concerns (treatment side effects), psychological concerns, priorities for treatment selection, and side effects to be avoided during treatment. Patients were asked about desired treatment information/information sources. Rankings were calculated using the mean value of scores. Spearman’s rank correlation was used to determine the concordance of rankings among groups.

**Results:**

Survey respondents included 207 patients, 185 physicians, and 150 nurses. Patients and nurses similarly ranked distressing physical concerns; physician rankings differed. Quality of life (QoL) and treatment response ranked high with physicians and patients when considering future treatment; nurses prioritized QoL. All three groups generally agreed on ranking of psychological concerns experienced during chemotherapy, explanation of treatment options, and how treatment decisions were made, although more patients thought treatment decisions should be made independently. Healthcare providers reported providing explanations of treatment side effects and information on physical/psychological support options while patients felt both were lacking. Concordance was calculated as 0.47 (patient–physician), 0.83 (patient–nurse), and 0.76 (physician–nurse). Patients desired additional information, preferring healthcare providers as the source.

**Conclusions:**

Specific areas for improvement in breast cancer patient care were identified; programs should be implemented to address unmet needs and improve treatment in these areas.

**Electronic supplementary material:**

The online version of this article (10.1007/s00520-019-05029-z) contains supplementary material, which is available to authorized users.

## Introduction

The progress made in breast cancer treatment over the last decade, including new diagnostic methods and approval of targeted therapeutic agents, has improved patient survival rates [1]. These treatment improvements have been observed for both early breast cancer (eBC) and metastatic breast cancer (mBC). Perceptions of the potential for survival of breast cancer in patients and healthcare professionals have also changed.

Regarding disease awareness in patients, a survey conducted in 2008 reported differences between major concerns of patients and those of physicians and nurses [2]. Fear of metastases, fatigue, consciousness of one’s own vulnerability, hair loss, and nausea ranked highest among patients [2]. Ratings of nurses and physicians strongly correlated with each other; however, ratings for some survey items showed a low correlation with patients [2]. Recent studies have reported discrepancies between the rankings of patients and physicians regarding the benefit of postoperative adjuvant chemotherapy [3, 4]. Other studies have identified unmet needs regarding hormone therapy–related side effects and long-term side effects [5, 6]. The most prominent unmet needs of patients were reported to be those in the psychological domain [7].

In Japan, the major target of the medical care system had been shifted to “reduction of burden among all cancer patients and their families and improvement of quality of life (QoL),” and “building a society in which cancer patients can live peacefully” [8]. However, it has been reported that there are still unmet information needs in younger patients with breast cancer [9]. There are few studies geared at detecting differences in perceptions related to breast cancer between these patients and doctors or nurses.

In the current therapeutic environment, it is clinically important to clarify the priority of the unmet medical information needs of breast cancer patients and to detect discrepancies in perceptions between physicians or nurses. This will maximize the effectiveness of communication between patients and healthcare professionals, making the best use of limited resources. We conducted a survey of physical and psychological perceptions toward treatment between breast cancer patients and healthcare professionals using an Internet questionnaire that was given to patients, physicians, and nurses in Japan.

## Materials and methods

### Study participants

For the survey, patients aged 20–69 years with a diagnosis of breast cancer who underwent chemotherapy within the past 5 years were recruited from a Medilead patient panel (Medilead, Inc., Tokyo, Japan). This general consumer panel consisted of 2 million people, 300,000 of whom have verified medical conditions and diseases, including cancer. From this panel, we contacted Japanese patients with breast cancer who met our study criteria. Physicians and nurses were recruited from the M3 panel (M3, Inc., Tokyo, Japan) which includes 250,000 physicians and 80,000 nurses in Japan. Doctors and nurses with a background suitable for this survey (i.e., had treated or cared for patients with breast cancer) received study questionnaires. Specifically, physicians (oncologists or surgeons) who belonged to a department of breast oncology/surgery, department of general surgery, or department of oncology, who had experience treating more than 10 breast cancer patients per month with chemotherapy, and nurses who had managed at least one breast cancer patient in the past month were recruited. Participant recruitment occurred between 14 May 2018 and 1 June 2018. Within this period, participants were recruited for each group until the target sample size for that group (200 patients, 200 physicians, 150 nurses) was reached. The number of participants was defined by the feasibility of each research panel.

### Measurements

An Internet survey aimed at thorough identification of circumstances surrounding common breast cancer treatments in Japan was implemented by Medilead, Inc. The survey questionnaire was developed with reference to previously published questionnaires [2, 10, 11] and was validated by doctors, nurses, pharmacists, and patients (Online Resource 1).

Background information and the following survey items were asked of all survey participants: (1) physical symptoms during chemotherapy, (2) psychological anxiety, (3) priorities at treatment selection, and (4) side effects considered most desirable to avoid when selecting treatment. Patients were surveyed regarding: (1) the most necessary information on breast cancer treatment (treatment-related), (2) the most necessary information on breast cancer treatment (other than treatment-related), and (3) desirable information sources and their usefulness.

Hormone receptor and HER2 status were assessed based on the American Society of Clinical Oncology–College of American Pathologists guideline definitions [12] in clinical settings.

### Statistical analysis

Answers to questions, other than single-choice questions, were rated on a 5-point scale, and rank was calculated using the mean value of scores. The concordance of rankings among the three groups was examined using Spearman’s rank correlation, and side effects and concerns regarding chemotherapy were cross tabulated with screening items. Statistical analyses were performed by Medilead, Inc.

### Ethical considerations

Study participants comprised individuals who responded to screening questions. Informed consent was obtained from all individual participants included in the study as part of the survey. Although no personal information was collected, disease information was carefully handled. Medilead, Inc. stored the results of the survey on a server housed inside Medilead, Inc.

## Results

### Participants

A total of 207 patients were included in the analyses. Their regional distribution reflected the general Japanese population. Mean age was 50.8 years (range 26–69 years), and approximately 80% of patients did not experience recurrence or metastasis (Table 1). Fifty-six patients (27.1%) were currently receiving chemotherapy; all others had a history of chemotherapy within the previous 5 years. Most patients received chemotherapy via intravenous infusion. Approximately 30% of patients received therapies targeting cell surface proteins and genes. Chemotherapeutic agents used in at least 10 patients were the following: docetaxel (*n* = 45); fluorouracil, epirubicin, and cyclophosphamide (FEC) (*n* = 33); paclitaxel (*n* = 29); nab-paclitaxel (*n* = 17); epirubicin and cyclophosphamide (EC) (*n* = 17); docetaxel and cyclophosphamide (TC) (*n* = 16); doxorubicin and cyclophosphamide (AC) (*n* = 10), all injections; and capecitabine (*n* = 12), an oral formulation.

**Table 1:** Background of patients, physicians, and nurses

	*n* (%)
Patients	*207*
Age	
30 years and younger	20 (9.7)
40 years	69 (33.3)
50 years	86 (41.5)
60 years and older	32 (15.5)
Breast cancer subtypes	
HR-positive/HER2-positive	69 (33.3)
HR-positive/HER2-negative	51 (24.6)
HR-negative/HER2-positive	28 (13.5)
HR-negative/HER2-negative	28 (13.5)
Unknown	31 (15.0)
Breast cancer status	
Operated, without recurrence/metastasis	162 (78.3)
Operated, with recurrence/metastasis	37 (17.9)
Unoperated, without recurrence/metastasis	3 (1.4)
Unoperated, with recurrence/metastasis	5 (2.4)
Drug therapy	
Chemotherapy	195 (94.2)
Hormonal therapy	132 (63.8)
Targeted therapy	61 (29.5)
Physicians	*185*
Departments	
Department of breast oncology/surgery	81 (43.8)
Department of general surgery	81 (43.8)
Department of oncology	23 (12.4)
Affiliated institutions	
University hospitals	45 (24.3)
Designated cancer care hospitals	54 (29.2)
General hospitals	78 (42.2)
Other	8 (4.3)
Nurses^a^	*150*
Hospital wards	67 (44.7)
General outpatient units	48 (32.0)
Outpatient chemotherapy units	45 (30.0)
Breast cancer outpatient units	13 (8.7)
Home visit nursing	6 (4.0)

Numbers in italics represent the total number of participants in each group

^a^Some nurses had more than one affiliation; hence, the numbers by affiliation type were summed to more than 150

A total of 185 physicians including those practicing in breast oncology/surgery (81 [43.8%]), general surgery (81 [43.8%]), and oncology (23 [12.4%]) responded to the survey. Affiliated institutions included university hospitals (24.3%), designated cancer care hospitals (29.2%), and general hospitals (42.2%) (Table 1).

A total of 150 nurses were included in the analyses. The nurses worked in hospital wards (67 [44.7%]), general outpatient units (48 [32.0%]), outpatient chemotherapy units (45 [30.0%]), and breast cancer outpatient units (13 [8.7%]). Some nurses had multiple affiliations (Table 1).

### Physical and psychological concerns

**Fig. 1:**
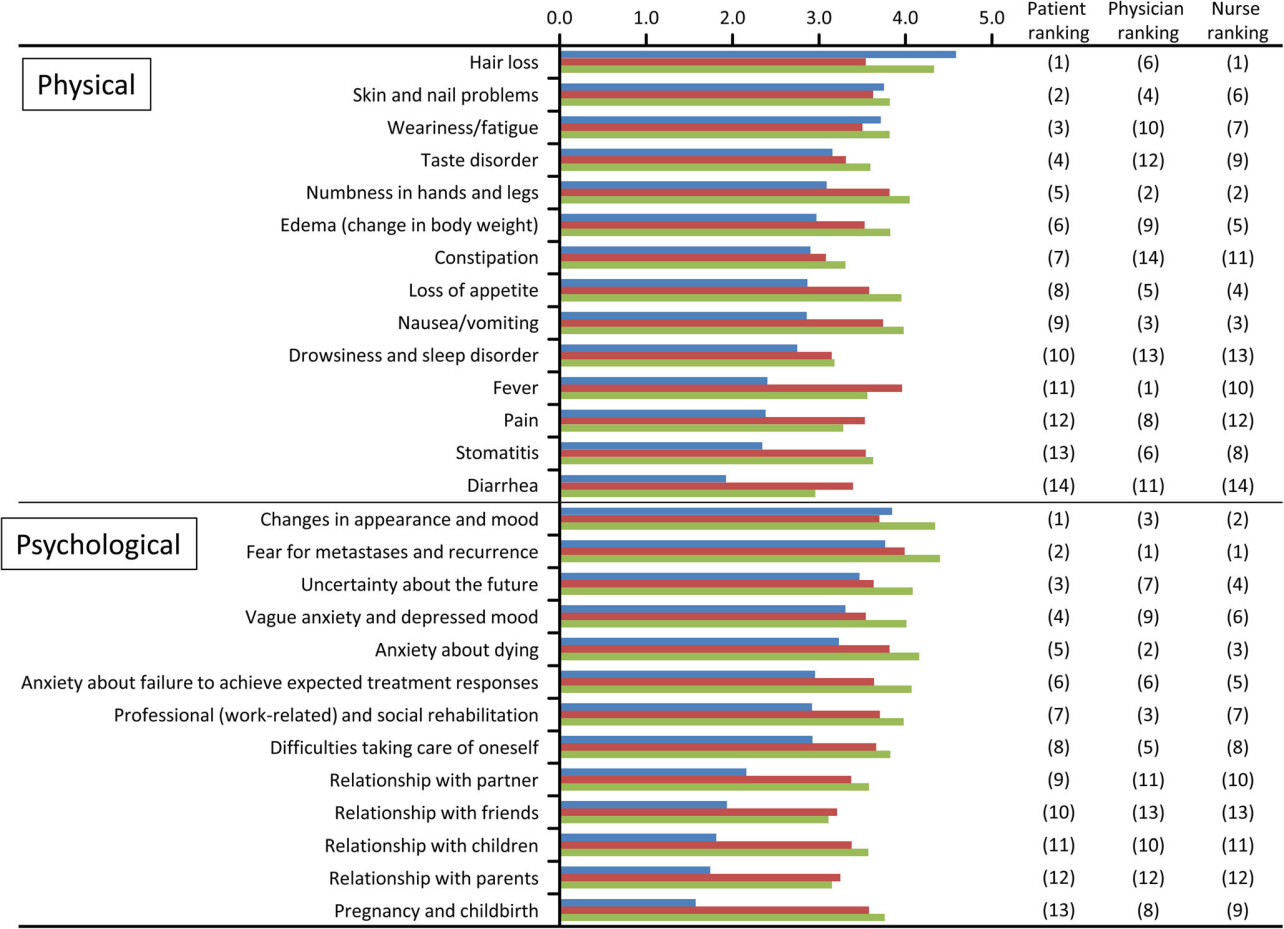
Ranking of the physical concerns (side effects) and psychological concerns experienced during breast cancer chemotherapy. Blue bar, patients; red bar, physicians; green bar, nurses

Regarding physical concerns (side effects) experienced by patients during chemotherapy, the most distressing was hair loss, followed by skin and nail problems, weariness/fatigue, taste disorder, numbness in hands and legs (peripheral neuropathy), and edema (Fig. 1). The severity of distress from nausea/vomiting was not high. Side effects most concerning to physicians were fever, followed by numbness in hands and legs (peripheral neuropathy), nausea/vomiting, skin and nail problems, loss of appetite, and hair loss. Nurses were most concerned about hair loss, also top-ranked by patients, followed by numbness in hands and legs (peripheral neuropathy), and nausea/vomiting, both of which were of high concern to physicians.

When asked about side effects most desirable to avoid when selecting treatment, nausea/vomiting and hair loss were ranked high by both patients and nurses. However, physicians most highly ranked pain, followed by nausea/vomiting, then fever; hair loss ranked low (data not shown). Skin and nail problems were a major concern for nurses but not for patients and physicians (data not shown).

Regarding psychological concerns experienced during chemotherapy, patients were highly concerned about “changes in appearance and mood,” “fear for metastases and recurrence,” “uncertainty about the future,” and “vague anxiety and depressed mood.” This was similar to the results of physicians and nurses (Fig. 1). Concerns about therapeutic response, such as “death and anxiety about dying” and “anxiety about failure to achieve expected treatment responses” were ranked similarly high in patients, physicians, and nurses.

**Fig. 2:**
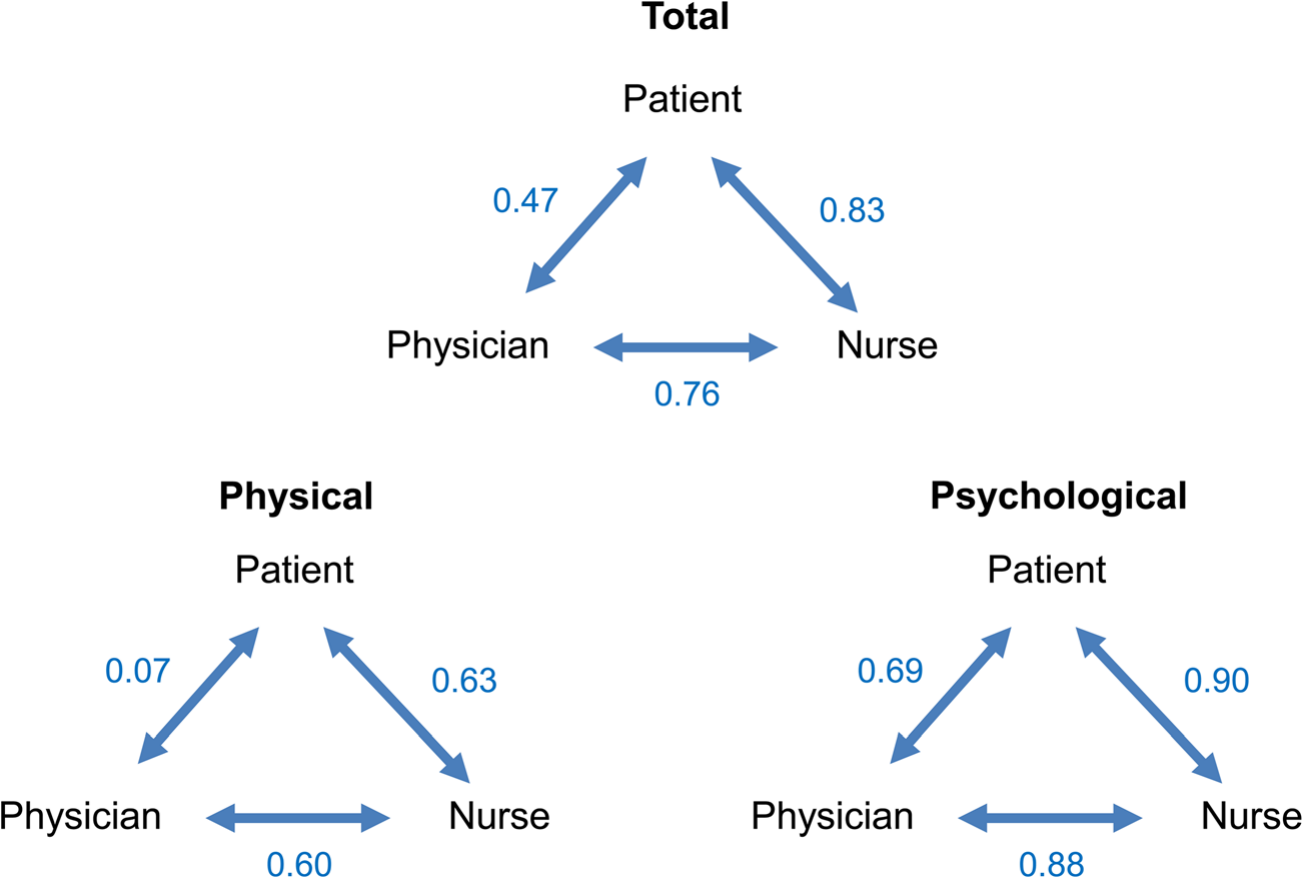
Rank correlation coefficients for physical concerns (side effects) and psychological concerns between patients, physicians, and nurses

The results of rank correlation for concern analyzed by Spearman’s rank correlation coefficient (Fig. 2) demonstrated that the correlation between patients and physicians was lower (0.47) than that between nurses and patients (0.83) and that between physicians and nurses (0.76). This was mainly caused by the significantly low correlation between patients and physicians (0.07) regarding physical concerns (side effects) and was greatly affected by the difference in ranking of “hair loss” between the two groups. The correlation in psychological concerns was relatively high between patients and physicians (0.69).

### Priority of treatment attributes for future treatments

For the questions regarding the priority of treatment attributes important in determining future treatments for the patient, approximately 47% of patients chose items related to QoL (QoL or mild side effects) and approximately 40% of patients chose items related to treatment response (longer survival or tumor reduction) (Online Resource 2). Approximately 51% and 47% of physicians chose items related to QoL and treatment response, respectively, showing a similar trend to patients. A high proportion (82%) of nurses chose items related to QoL.

### Information received or expected to have been received by patients

**Fig. 3:**
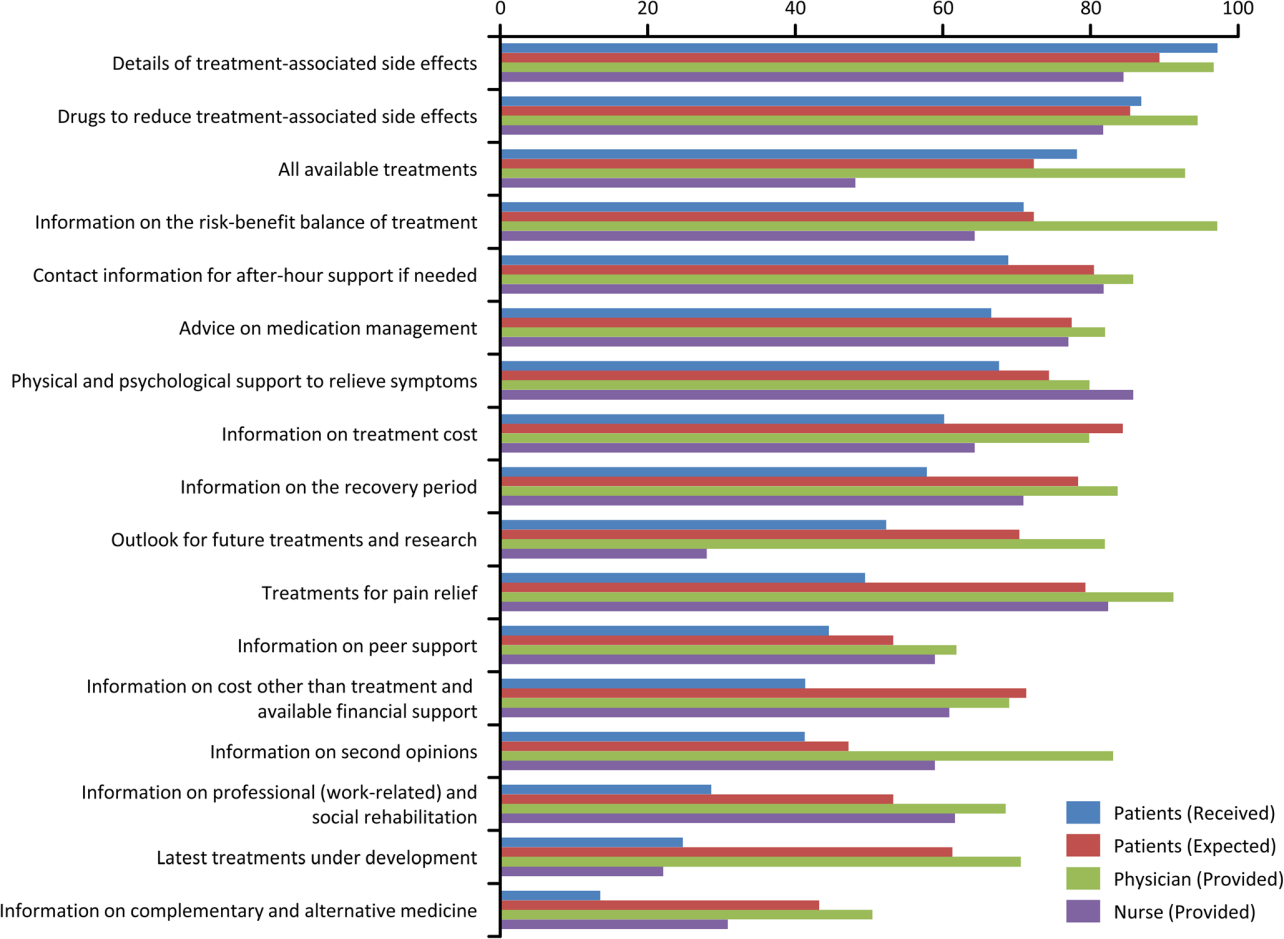
Treatment information for which an explanation was considered as having been received/provided/expected. Results are shown for each survey group and information item and are represented as a percentage of the total number of responses. Answers chosen by < 3% of respondents are not listed

Information related to chemotherapy that patients perceived as having been received included “treatment-associated side effects” (97%), “drugs to reduce treatment-associated side effects” (86%), “all available treatments” (78%), and “risk-benefit balance of treatment” (71%). These informational items were all expected to be received by patients and perceived as provided by physicians (Fig. 3). However, some informational items were expected to be received by patients but were perceived as not having been received. These items included information on “treatment cost” (expected 84% vs received 60%), “recovery period” (78% vs 57%), “outlook for future treatments and research” (52% vs 70%), “treatments for pain relief” (79% vs 49%), “cost other than treatment and available financial support” (71% vs 41%), and “professional (work-related) and social rehabilitation” (53% vs 29%). These informational items were perceived as having been *provided* by most physicians and nurses.

### Treatment decision-making

Most physicians and nurses thought that treatment decisions should be made either through discussion between patients and physicians or by patients with consideration of the opinion of their treating physician. However, a higher percentage of patients (25%) thought that treatment should be determined by physicians (data not shown). This trend was higher in elderly patients (data not shown).

Regarding the most desirable person for consultation regarding chemotherapy, most patients thought that the physician was the only person who should be consulted (Online Resource 3). However, physicians and nurses both wanted patients to seek consultations with nurses and pharmacists in addition to physicians.

## Discussion

The present survey revealed that there are still gaps in perceptions between healthcare professionals and patients, despite recent progress in breast cancer treatment. Although the rankings for severity of psychological concerns were correlated between patients and nurses, and between patients and physicians, large differences were observed in the rankings of physical concerns (side effects) between patients and physicians.

Regarding physical concerns, “hair loss” and “skin and nail problems” were highly ranked by patients as both the most distressing chemotherapy side effects and most desirable to avoid when selecting treatment, but these were ranked as less important by physicians. This result was reflected in the higher ranking of “changes in appearance (hair loss and complexion) and mood” in patients and nurses regarding psychological anxiety. With an improved survival rate for breast cancer [1], more patients continue to work while undergoing chemotherapy, which increases the importance of treatment-related appearance changes for patients.

Distress caused by changes in appearance is reported to be particularly intense in patients with breast cancer compared with patients with other types of cancer [13]. For physicians, hair loss was not a major concern because it is not a life-threating issue and, historically, there has been no effective approach to manage it. However, scalp cooling has recently been shown to prevent chemotherapy-induced hair loss [14, 15]. These findings may result in increased efforts for the management of hair loss. In Japan, the concept of “appearance care” was recently proposed by the National Cancer Center Hospital. As we shift to an era focused on the management of cancer as chronic diseases (in which the need for both a cure and improved care are recognized), we should be aware that changes in appearance significantly affect the QoL of many patients despite their lack of impact on life expectancy. It is therefore essential to establish a system that allows patients easy access to appearance support.

Contrary to the concerns of patients and nurses, physicians perceived the most important side effects for concern as fever, numbness in hands and legs (peripheral neuropathy), and pain. Physicians consider these of high concern because fever may be a sign of serious complication and peripheral neuropathy may cause a dose reduction or discontinuation of chemotherapy. Nausea/vomiting was also recognized as important to manage because it can cause a reduction in both QoL and the continuation of chemotherapy. As a result of the high priority placed on the management of nausea/vomiting by physicians and nurses, these side effects are relatively controlled in clinical practice. The resulting decreased occurrence may have led to reduced concerns in patients.

Regarding psychological concerns experienced during chemotherapy, patients were highly concerned about “changes in appearance and mood.” We speculate this may be related to hair loss; this point differed from the previous survey [2].

Regarding treatment efficacy, concerns of “fear for metastases and recurrence,” “anxiety about dying,” and “anxiety about failure to achieve expected treatment responses” were ranked similarly high by patients, physicians, and nurses. In other words, these concerns might reflect longer OS as an attribute for future treatment and were consistent with the results of the previous survey [2]. In the present survey, the proportion of patients with recurrent or metastatic cancer was 20%. In Japan, breast cancer mortality has plateaued in recent years [16]. Although targeted agents have become available, advanced breast cancer is still considered treatable but not curable. A national survey to understand the attitudes of oncologists toward end-of-life discussions in Japan indicated that oncologists should reflect on their own values and knowledge to help them manage and improve the facilitation of end-of-life discussions [17]. It is expected that timely end-of-life discussions between oncologists and patients will help reduce the level of “anxiety about dying” identified by our study.

Regarding the difference between the information which patients perceived they had received and the information that was necessary for them to receive (information that should be provided), the core therapeutic information (risk and benefit of chemotherapy) was considered as necessary information, as information having been received by patients, and as information considered by physicians and nurses to have been provided to patients. However, patients felt there was a shortage of information regarding cost (treatment or other), pain relief, professional and social rehabilitation, and information provided during their recovery period despite the fact that patients felt this information should be provided to them. The types of information perceived to be lacking by the patients suggest that patients realized the need for this information after receiving chemotherapy.

Over half of physicians and nurses believed they had fully explained physical and psychological support available to relieve symptoms, but patients reported that the information was inadequate. “Provision of palliative care from the time of diagnosis” is a mainstay of cancer control so it may be necessary to provide this information through multiple channels, encouraging patients to use palliative care and to liaise with palliative care specialists wherever possible. This study also showed that there is a need for better provision of information regarding the use of the social security system for help with treatment costs, particularly because the agents used to treat breast cancer are known to be expensive [18].

The discrepancy between physician and nurse beliefs around their explanations of the support available and patient perceptions about these explanations could arise because the explanations really are insufficient. Alternatively, the mental state of the patient at the time of explanation could impact how well an explanation is understood. Regarding information other than that on treatment, patients strongly wished to obtain “information on how to communicate with healthcare professionals,” further suggesting that patients may have felt there was a communication gap. In terms of health information, relationships between health literacy and physical/mental activity have been reported [19, 20]. This health literacy refers to the ability of a patient to recognize the symptoms of physical and mental illness and be aware of both self-treatment and professional help available for these disorders. Efforts to enhance health literacy may help to bridge the communication gap between patients and healthcare professionals.

Regarding consultation for the choice of chemotherapy, patients thought that physicians were both the most appropriate and most important person to consult. Both physicians and nurses expressed a desire for patients to consult nurses and pharmacists more actively. This suggests that while healthcare professionals understand that nurses and pharmacists can provide information regarding the management of side effects, patients do not know that consulting with nurses and pharmacists is a viable option.

Our study had several limitations resulting from the use of a questionnaire survey. These limitations included that the responses were chosen from a list, therefore limiting responses; the questionnaire required the time and effort of respondents; there was a possible bias in that respondents may not have disclosed inconvenient information; and there was uncertainty about the credibility of answers received. The survey respondents may have been limited to those who are highly conscious of breast cancer treatment.

## Conclusion

The results of this survey revealed some differences between patients and physicians in the perception of chemotherapy. While healthcare providers believed they were appropriately sharing information with patients, patients felt that they had not received adequate information regarding pain relief, treatment side effects, and options for physical and psychological support. Both patients and physicians were highly aware of the importance of shared decision-making about treatment selection and of the provision of necessary basic information for decision-making, suggesting that the overall awareness of these points in Japan is good.

Specific areas to focus on for improving overall communication between healthcare providers and patients include the establishment of educational programs to inform healthcare professionals that more patients may need appropriate informational and psychological support. Healthcare providers should strive to provide a wide range of information and take time to fully understand and address the sentiments of patients regarding their treatment and care.

## Electronic supplementary material

Online Resource 1 Questionnaire completed by participants. (PDF 94 kb)

Online Resource 2 Priority of treatment attributes for the future treatment of breast cancer. Answers chosen by < 3% of respondents are not listed. QoL: quality of life. (PDF 314 kb)

Online Resource 3 Preferred source of consultation regarding breast cancer chemotherapy. Patients with breast cancer who had received chemotherapy within the past 5 years, physicians (oncologists or surgeons) who had treated patients with breast cancer and nurses who had at least one breast cancer patient in their care were surveyed and asked who they considered to be the preferred source of consultation regarding breast cancer chemotherapy. Results are shown for each survey group and are represented as a percentage of the total responses. (PDF 320 kb)
