# Supplementary material for: Differences in perception of breast cancer treatment between patients, physicians, and nurses and unmet information needs in Japan
Source: Support Care Cancer. 2019 Sep 3;28(5):2331–8. doi: 10.1007/s00520-019-05029-z (PMC7083820; doi:10.1007/s00520-019-05029-z)
Supplement: Supplementary file 1 — Questionnaire completed by participants. (PDF 94 kb) [file 520_2019_5029_MOESM1_ESM.pdf]

Online Resource 1.

Q1. Please tell us about the following physical symptoms you have felt during your previous chemotherapy for breast cancer.
For each, answer "1" for "no symptoms" and "5" for "most severe symptoms" for "symptoms."

1	Pain
2	Nausea and vomiting
3	Anorexia
4	Diarrhea
5	Constipation
6	Taste disorder
7	Stomatitis
8	Hair loss
9	Skin and nail problems
10	Edema (change in body weight)
11	Numbness of hands and feet (peripheral neuropathy)
12	Fever
13	Weariness and fatigue
14	Drowsiness and sleep problems
15	Other ( )

Q2. Please describe the intensity you have felt during your previous chemotherapy for breast cancer about the following life anxiety and concerns.
For each, please answer "1" for "no worry" and "5" for "most worried."

1	Vague anxiety and depressed mood
2	Anxiety that the results of treatment will not be as expected
3	Uncertainty in the future
4	Fear of metastasis/recurrence
5	Changes in relationship with partner
6	Changes in relationship with parents
7	Change in relationship with children
8	Change in relationship with friends
9	Difficulties taking care of oneself
10	Concern and anxiety about employment and rehabilitation
11	Changes in appearance and atmosphere (hair loss and facial color)
12	Concern and anxiety about pregnancy and childbirth
13	Anxiety about dying or dying
14	Other ( )

Q3. Please assume that pharmacological treatment for breast cancer may be added in the future. If your doctor suggests more than one treatment option (drug treatment), what features of your drug treatment do you prefer to choose? Please inform us of the top three. Please also tell us one of the most priority features.

1	Longer survival
2	Cancer reduction
3	Can live while maintaining quality of life (QoL)
4	Mild side effects
5	Fewer treatment hours
6	Can be treated on an outpatients basis
7	Inexpensive treatment cost
8	Other (　　　　)
9	There are no preferential features.

Q4. If you assume that you will receive drug treatment for breast cancer in the future, please inform us the following three side effects if you can avoid them. Please also tell us one of the side effects you want to avoid.

1	Pain
2	Nausea and vomiting
3	Loss of appetite
4	Diarrhea
5	Constipation
6	Taste disorder
7	Stomatitis
8	Hair loss
9	Skin and nail problems
10	Edema (change in body weight)
11	Numbness of hands and feet (peripheral neuropathy)
12	Fever
13	Weariness and fatigue
14	Sleeping difficulties
15	Other (　　　　)
