# Supplementary material for: Differences in perception of breast cancer treatment between patients, physicians, and nurses and unmet information needs in Japan
Source: Support Care Cancer. 2019 Sep 3;28(5):2331–8. doi: 10.1007/s00520-019-05029-z (PMC7083820; doi:10.1007/s00520-019-05029-z)

Online Resource 2

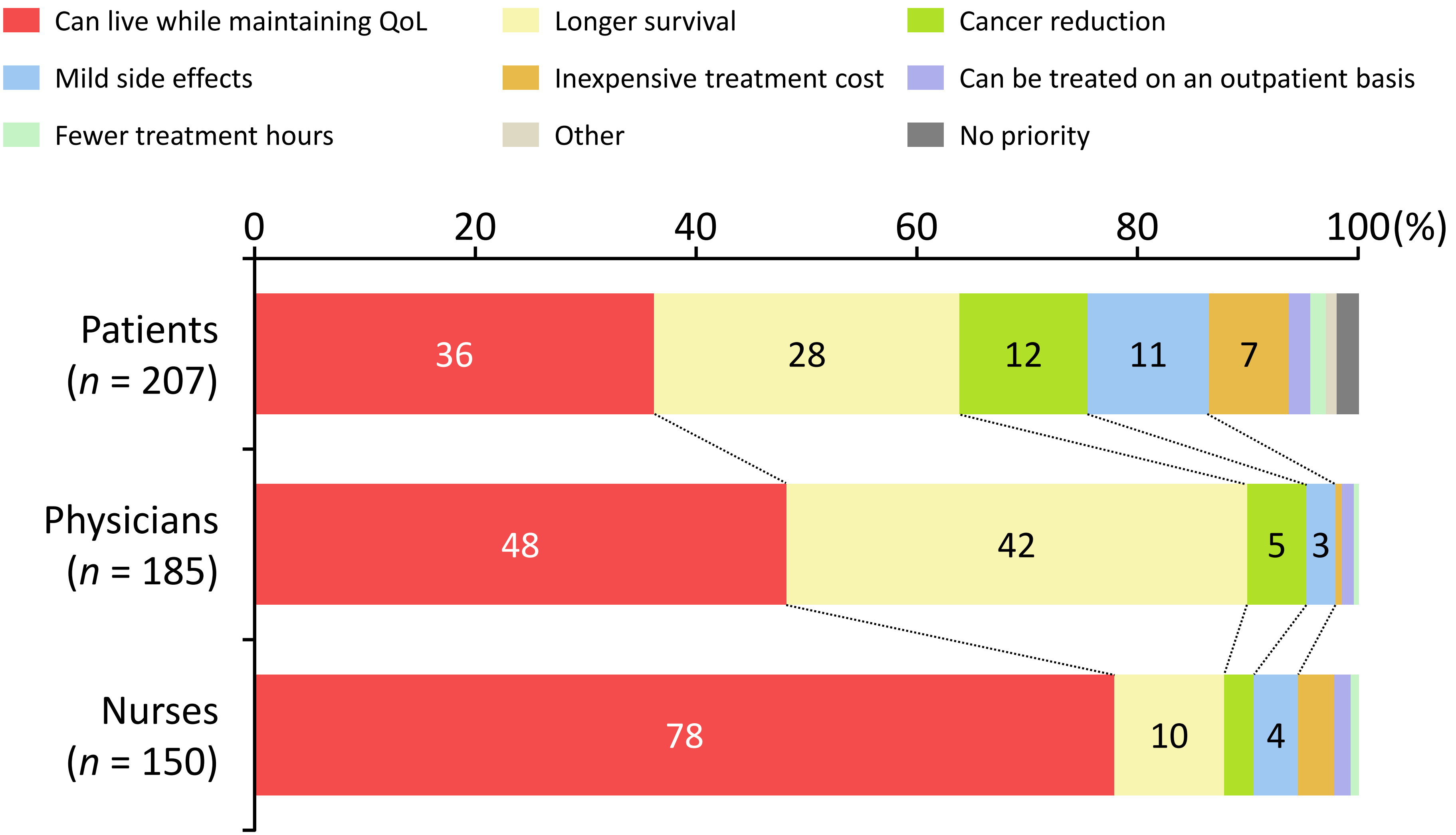

Supplement: Supplementary file 2 — Priority of treatment attributes for the future treatment of breast cancer. Answers chosen by < 3% of respondents are not listed. QoL: quality of life. (PDF 314 kb) [file 520_2019_5029_MOESM2_ESM.pdf]
