# Supplementary figures and images for: Differences in perception of breast cancer treatment between patients, physicians, and nurses and unmet information needs in Japan
Source: Support Care Cancer. 2019 Sep 3;28(5):2331–8. doi: 10.1007/s00520-019-05029-z (PMC7083820; doi:10.1007/s00520-019-05029-z)

Online Resource 3

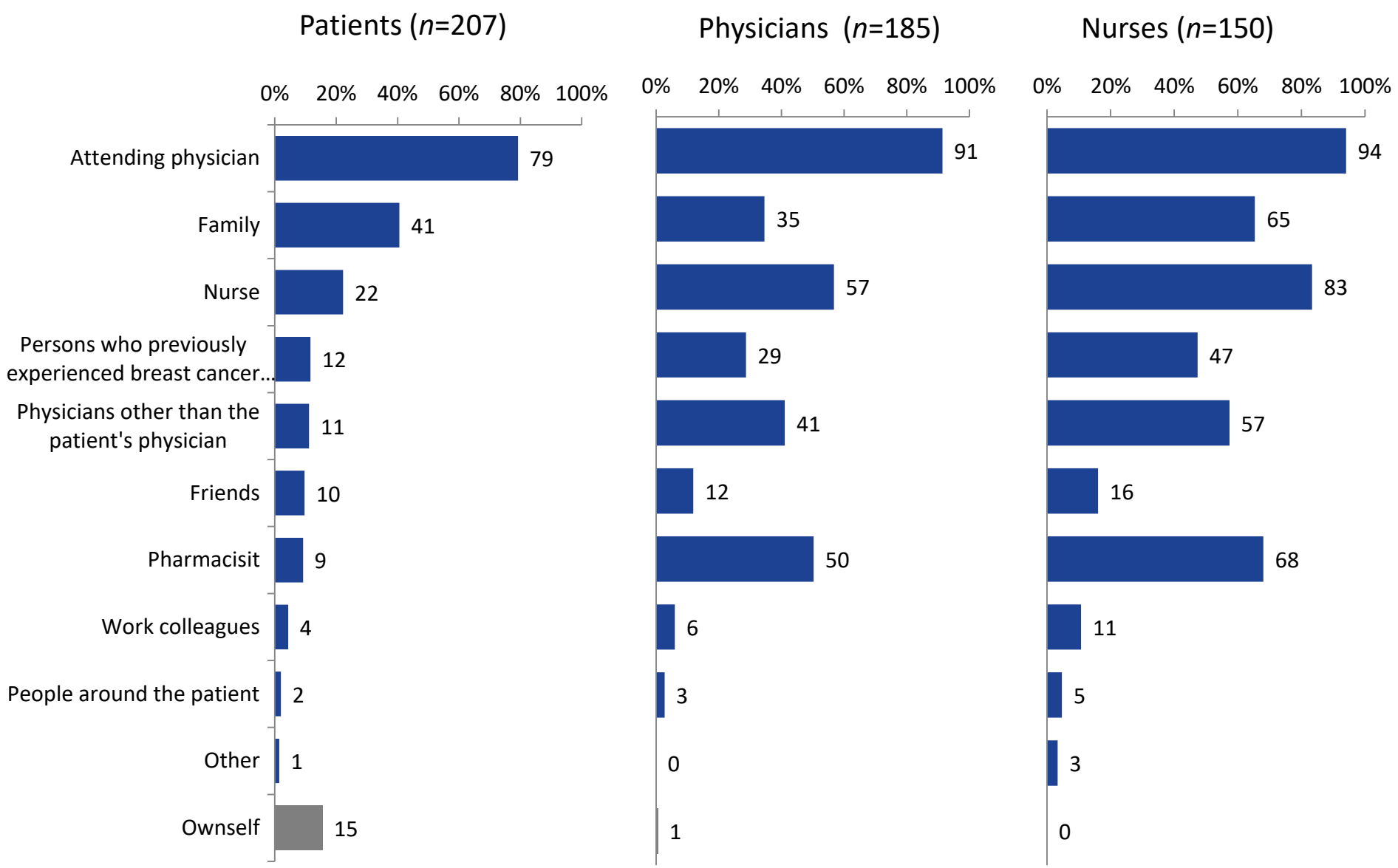

Supplement: Supplementary file 3 — Preferred source of consultation regarding breast cancer chemotherapy. Patients with breast cancer who had received chemotherapy within the past 5 years, physicians (oncologists or surgeons) who had treated patients with breast cancer and nurses who had at least one breast cancer patient in their care were surveyed and asked who they considered to be the preferred source of consultation regarding breast cancer chemotherapy. Results are shown for each survey group and are represented as a percentage of the total responses. (PDF 320 kb) [file 520_2019_5029_MOESM3_ESM.pdf]
